# Correlation of multimodal ^18^F-DOPA PET and conventional MRI with treatment response and survival in children with diffuse intrinsic pontine gliomas

**DOI:** 10.7150/thno.50598

**Published:** 2020-10-25

**Authors:** Giovanni Morana, Domenico Tortora, Gianluca Bottoni, Matteo Puntoni, Gianluca Piatelli, Federica Garibotto, Salvina Barra, Flavio Giannelli, Angelina Cistaro, Mariasavina Severino, Antonio Verrico, Claudia Milanaccio, Maura Massimino, Maria Luisa Garrè, Andrea Rossi, Arnoldo Piccardo

**Affiliations:** 1 Neuroradiology Unit, IRCCS Istituto Giannina Gaslini, Genova, Italy.; 2 Department of Neurosciences, University of Turin, Turin, Italy.; 3 Nuclear Medicine Unit, Ente Ospedaliero Ospedali Galliera, Genova, Italy.; 4 Clinical Trial Unit, Scientific Directorate, Ente Ospedaliero Ospedali Galliera, Genoa, Italy.; 5 Neurosurgery Unit, IRCCS Istituto Giannina Gaslini, Genova, Italy.; 6 Neuro-oncology Unit, IRCCS Istituto Giannina Gaslini, Genova, Italy.; 7 Department of Radiation Oncology, Ospedale Policlinico San Martino and University, Genova, Italy.; 8 Pediatric Unit, Fondazione IRCCS Istituto Nazionale dei Tumori, Milano, Italy.

**Keywords:** DOPA PET, MRI, Pediatric, DIPG, diffuse midline glioma

## Abstract

To evaluate the contribution of ^18^F-dihydroxyphenylalanine (DOPA) PET in association with conventional MRI in predicting treatment response and survival outcome of pediatric patients with diffuse intrinsic pontine gliomas (DIPGs).

**Methods:** We retrospectively analyzed 19 children with newly diagnosed DIPGs who underwent ^18^F-DOPA PET/CT and conventional MRI within one week of each other at admission and subsequent MRI follow-up. Following co-registration and fusion of PET and MRI, ^18^F-DOPA uptake avidity and extent (PET tumor volume and uniformity) at admission, along with MRI indices including presence of ring contrast-enhancement, tumor volume at admission and at maximum response following first-line treatment, were evaluated and correlated with overall survival (OS). The association between ^18^F-DOPA uptake tumor volume at admission and MRI tumor volume following treatment was evaluated. Statistics included Wilcoxon signed-rank and Mann-Whitney U tests, Kaplan-Meier OS curve and Cox analysis.

**Results:** DIPGs with a ^18^F-DOPA uptake Tumor/Striatum (T/S) ratio >1 presented an OS ≤ 12 months and lower degree of tumor volume reduction following treatment (*p* = 0.001). On multivariate analysis, T/S (*p =* 0.001), ring enhancement (*p =* 0.01) and the degree of MRI tumor volume reduction (*p =* 0.01) independently correlated with OS. In all patients, areas of increased ^18^F-DOPA uptake overlapped with regions demonstrating more prominent residual components/lack of response following treatment.

**Conclusions:** ^18^F-DOPA PET provides useful information for evaluating the metabolism of DIPGs. T/S ratio is an independent predictor of outcome. ^18^F-DOPA uptake extent delineates tumoral regions with a more aggressive biological behaviour, less sensitive to first line treatment.

## Introduction

In pediatric patients, diffuse intrinsic pontine glioma (DIPG) represents the most difficult brain tumor to treat and the leading cause of brain tumor-related death 1,2.

Despite recent remarkable genomic discoveries, partially included in the revised 2016 WHO classification, such as highly recurrent H3K27M histone mutations 3, there has been no significant progress in the management of DIPG. Radiotherapy (RT) remains the current standard of care, providing transient clinical improvement and a limited survival benefit. Tumor progression is almost universal, with median overall survival less than 1 year 4. Several clinical trials over the last few decades have also investigated different adjuvant chemotherapies (ChT), albeit without significant survival benefit 4-6. Routine biopsy in DIPG remains under debate 7 since the diagnosis may be established with imaging alone 8.

Several prior imaging studies focusing on conventional and/or advanced MRI modalities have attempted to identify non-invasive parameters able to predict survival at the time of diagnosis with not univocal results, most requiring further investigations in large DIPG clinical trials 9-14.

Additional non-invasive biomarkers that could predict disease evolution are still awaited to contribute to the establishment of well-timed, personalized and more effective therapies, aiming to improve overall survival and quality of life of DIPG patients.

Positron Emission Tomography (PET) imaging with amino-acid tracers, such as ^18^F-dihydroxyphenylalanine (DOPA), is a diagnostic tool able to provide non-invasive information of brain tumors. ^18^F-DOPA uptake has been demonstrated to correlate with aggressiveness of diffusely infiltrating pediatric gliomas 15-17 and with H3K27M mutational status, independently of histology 18. The activity of ^18^F-DOPA PET seems both based on and strictly correlated to tumor metabolism, which is associated with enhanced and altered channeling of amino-acids for protein synthesis and cell division in pediatric high-grade infiltrative astrocytomas 19. No prior studies have so far evaluated metabolic information obtained by ^18^F-DOPA PET focusing on DIPGs.

On the basis of these considerations, the overall objective of this retrospective study was to analyze diagnostic and prognostic information obtained by ^18^F-DOPA PET in a group of children with newly diagnosed DIPGs. Specifically, we aimed to evaluate the contribution of the ^18^F-DOPA PET delineated tumor volume at diagnosis to conventional MRI based tumor volume at diagnosis and at maximum response following first line ChT-RT treatment, and to correlate ^18^F-DOPA PET and MRI indices with outcome in terms of overall survival (OS).

## Methods

### Patient population

We retrospectively evaluated all consecutive pediatric patients (aged less than 18 years at diagnosis) referred at IRCCS Istituto Giannina Gaslini, Genoa, Italy between 2012 and 2019 for newly diagnosed treatment naïve DIPGs (T1 hypointense and T2 hyperintense diffusely infiltrating lesion arising and involving ≥ 50% of the pons) who underwent conventional MRI and ^18^F-DOPA PET at admission, and subsequent post-treatment MRI follow-up.

Nineteen (12 females and 7 males) subjects were identified. Patient age ranged from 3 to 10 years (median, 7 years). Six of these patients had been previously included in a retrospective study aimed to evaluate the diagnostic ability of ^18^F-DOPA PET and advanced MRI techniques in discriminating H3K27M-mutant from wild-type pediatric diffuse midline gliomas 18.

Clinical information reviewed for each subject included the time of diagnosis and treatment onset, OS (defined as the interval from treatment initiation to death from any cause), and treatments received. Because OS is regarded as the most reliable outcome variable for DIPG, progression-free survival was not evaluated 6.

Regarding treatment, all patients underwent first-line treatment with combined chemotherapy/immunotherapy (Vinorelbine and Nimotuzumab) and radiotherapy. Irradiation was performed in all patients using conventional fractionation (1.8 Gy per day) to a total dose of 54 Gy. In case of local progressive disease or progression with dissemination, re-irradiation was performed with variable doses and schedule according to disease sites (12 subjects underwent re-irradiation).

Ten patients underwent biopsy at diagnosis (following 18F-DOPA PET and MRI) with histological and molecular characterization. In particular, molecular analyses were performed to test the presence of mutations in the histone variants H3.3 (H3F3A) and H3.1 (HIST1H3B). Among subjects who underwent histological and molecular characterization, there were 2 patients with H3K27M wild-type (1 histologically defined diffuse astrocytoma and 1 anaplastic astrocytoma) and 8 with H3K27M-mutant lesions (4 histologically defined anaplastic astrocytomas and 4 glioblastomas). All subjects with H3K27M-mutant DIPGs presented mutations in the histone variant H3.3 (H3F3A).

Surveillance was performed with regular clinical and MRI follow-up. The Regional Ethics committee of Liguria, Genoa, Italy, approved the retrospective data evaluation.

### Image protocol and analysis

Because the study spanned 7 years, MRI examinations were performed on 1.5T (14 patients; Intera Achieva; Philips, Best, the Netherlands) and 3T (5 patients; Ingenia Cx, Philips, Best, the Netherlands) scanners.

All patients underwent routine clinical MRI examinations including axial fluid attenuation inversion recovery (FLAIR), T2-weighted images, and pre- and post-contrast (0.1 mmol/kg, macrocyclic ionic agent) T1-weighted images.

^18^F-DOPA PET/CT was carried out at admission within one week of baseline MRI. Imaging studies were performed within 2 weeks before treatment initiation; subsequent evaluations were performed with MRI at 4-5 weeks after RT completion and then every 12-13 weeks, unless new symptoms occurred. In case of suspected pseudo-progression at first post-treatment MRI evaluation, additional close MRI follow-up at 4-5 weeks was performed (2 patients).

^18^F-DOPA PET studies were executed with a PET/CT Discovery ST system (GE Healthcare, Milwaukee, WI, USA), as previously described 17,18. Data were acquired in 3-dimensional mode, 20 minutes after ^18^F-DOPA administration (median injected activity of 100 MBq, range 70-120 MBq according to body weight) with a scanning time of 30 minutes. Patients fasted for at least 4 hours before ^18^F-DOPA administration (IASOdopa®, IASON Labormedizine Ges. Mbh & Co. KG, Graz-Seiersberg, Austria). Carbidopa premedication was not utilized. A non-diagnostic low dose CT scan (120 kV, 80 mA, 0.6 s per rotation) was used for attenuation correction.

Images were first analysed on a dedicated workstation (Xeleris, GE Corporation), also allowing semiautomatic co-registration and fusion of ^18^F-DOPA PET and MR images to ensure precise anatomical comparability. Using dedicated software developed for research purposes (Quanta Oncology, Camelot Biomedical Systems, Genoa, Italy) we performed volumetric tumor analysis on axial ^18^F-DOPA PET and MRI FLAIR images. In detail, the anatomic tumor extent was delineated on MRI FLAIR images 20 using a perimeter technique with user-assisted semi-automated software; ^18^F-DOPA PET tumor volume was delineated based on ^18^F-DOPA uptake avidity (tumoral areas with increased uptake compared to normal background reference region). For each case tumor volume delineation was reviewed in consensus by a nuclear medicine physician and a neuroradiologist with 17 and 15 years of experience (A.P. and G.M.). Volume of interests (VOIs) were generated and ^18^F-DOPA PET and MRI tumor volume at admission were then recorded. The same procedure was performed following first-line treatment with ChT-RT on the MRI studies demonstrating the maximum degree of response (in all but two subjects the first MRI evaluation following ChT-RT was selected). MRI tumor volume following treatment and the corresponding tumor volume change since admission, were recorded.

For each patient, ^18^F-DOPA uniformity, defined as the percentage of the MRI tumor volume at admission (as delineated on FLAIR images) demonstrating increased ^18^F-DOPA PET uptake, was also calculated 21,22.

PET tumor volume was delineated on ^18^F-DOPA PET studies by including all voxels with standardized uptake value (SUV) above the maximum (max) SUV of the normal background reference tissue. For the normal (N) background reference tissue, a VOI (diameter 20 mm) was drawn in the normal cerebral hemisphere at the level of the left centrum semiovale, including cortical and white matter. For each case, the radiotracer concentration in the tumoral VOI was normalized to the injected dose per patient body weight, and the SUV max was obtained for each lesion [maximum pixel value (kBq/mL) within the VOI/injected dose (kBq)/patient weight (g)]. An additional VOI was drawn over the left striatum including the entire putamen (S). Ratios of tumor to normal tissue uptake were also generated by dividing the tumor SUVmax by the SUVmax of the striatum (T/S) 17,18. In case of absence of increased ^18^F-DOPA uptake, a VOI including the tumor on MRI FLAIR images was delineated on co-registered ^18^F-DOPA PET images to generate T/S and T/N ratios.

Three main tumor uptake patterns were defined: (i) absence of increased ^18^F-DOPA uptake, characterized by a tumor uptake not exceeding the uptake of the normal background reference tissue (T/N ≤ 1) and lower uptake than striatum (T/S < 1); (ii) mildly/moderately increased ^18^F-DOPA uptake, characterized by a tumor uptake exceeding the uptake of the normal background reference tissue but remaining lower than or equal to that of the striatum (T/N > 1 and T/S ≤ 1); and (iii) markedly increased ^18^F-DOPA uptake, in which tumor uptake clearly exceeded that observed in the normal background reference tissue and striatum (T/N > 1 and T/S > 1) 23.

For each patient we also evaluated the association between baseline ^18^F-DOPA PET avidity and MRI based tumor volume at maximum response by delineating VOIs and assessing the overlap of the residual MRI tumor volume and initial ^18^F-DOPA PET volume 22.

Presence or absence of areas of ring-shaped enhancement on post-contrast T1-weighted images was also recorded. Distribution of areas of ring-shaped enhancement in relation to areas of increased ^18^F-DOPA uptake was also evaluated on baseline MRI studies.

### Risk stratification

Each patient was risk stratified according to age, sex, H3K27M mutation, presence or absence of ring-shaped enhancement on MRI, pre- and post-treatment MRI tumor volume, 18F-DOPA uptake avidity (T/S), pre-treatment PET tumor volume and pre-treatment PET uniformity.

### Statistical analysis

Descriptive statistics included mean, standard deviation, minimum, and maximum of continuous factors and scores; in the case of categorical factors, number and percentage distribution were used.

Due to the non-normality of data (graphically checked), the Wilcoxon signed-rank test was used to evaluate pre- versus post-first line treatment changes among variables of interest, whereas the Mann-Whitney U tests was applied to compare MRI tumor volume changes after treatment between patients with and without increased ^18^F-DOPA uptake.

Kaplan-Meier estimates of the cumulative probability of OS at 12 months were performed and log-rank test was adopted to test differences between groups (log-rank test for trend in case of 3 or more ordinal groups). T/S was evaluated as a dichotomous variable (≤1 and >1) in the Kaplan-Meyer analysis. The Cox proportional hazard model was used to estimate the risk of death from any cause after adjustment for age and sex. Only the covariates that were significant at the *p* < 0.05 level in the univariate analysis (where we examined all risk factors included in our study), were entered into the multivariate analysis. The proportional hazard assumption was graphically checked. Since the parameters were highly correlated, to avoid collinearity, we used different models for each parameter to test their independent association with OS. We adopted 1/Variance Inflation Factor (VIF) as a measure of collinearity. Two-tailed probabilities were reported, and a *p* value of 0.05 was used to define nominal statistical significance; given the explorative nature of the study, no multiple testing corrections were applied. All analyses were conducted using Stata (version 13, Stata-Corp) software.

## Results

### Diagnostic evaluation and correlation with tumor response

Demographic data, histological and molecular diagnosis, MRI findings (ring enhancement, pre- and post-treatment MRI tumor volumes), ^18^F-DOPA uptake avidity and extent (PET tumor volume and uniformity), and survival of all patients are summarized in **Table 1.** A detailed report of all patients is provided in Table S1.

On baseline MRI, areas of ring-shaped enhancement were present in 6 DIPGs (31.6%). Three of these lesions were glioblastomas, H3K27M-mutant. The remaining 5 patients with H3K27M-mutant and the 2 patients with H3K27M wild-type DIPGs did not show areas of ring enhancement.

On ^18^F-DOPA PET imaging, 4 tumors (21%) showed absence of increased tracer uptake. Of these, two underwent biopsy and turned out to be H3K27M wild-type. Two DIPGs presented mildly/moderately increased tracer uptake (10.5%), whereas the remaining 13 lesions (68.5%) exhibited markedly increased uptake. All subjects with H3K27M-mutant DIPGs presented markedly increased tracer uptake (median T/S ratio: 1.49, range: 1.10-2.32). All subjects with ring-shaped enhancement on post-contrast T1-weighted images presented also markedly increased uptake. In detail, all areas of ring-shaped enhancement corresponded to regions with markedly increase uptake; however, markedly increase uptake extended beyond these areas in 5 out of 6 subjects.

No lesions presented ^18^F-DOPA PET tumor volumes larger than MRI defined tumor volumes or uptake extending beyond MRI defined tumor margins.

Following first line ChT-RT, MRI tumor volumes at maximum response were lower than pre-treatment tumor volumes (median 20 cc vs 27.64 cc, *p* < 0.001).

Patients with absence of increased tracer uptake and the 2 patients with mildly/moderately increase uptake (T/S ≤ 1) demonstrated higher median degree of MRI tumor volume reduction compared to subjects with markedly increased tracer uptake (T/S >1) (23.5 cc versus 5.52 cc; *p =* 0.001).

**Figure 1:**
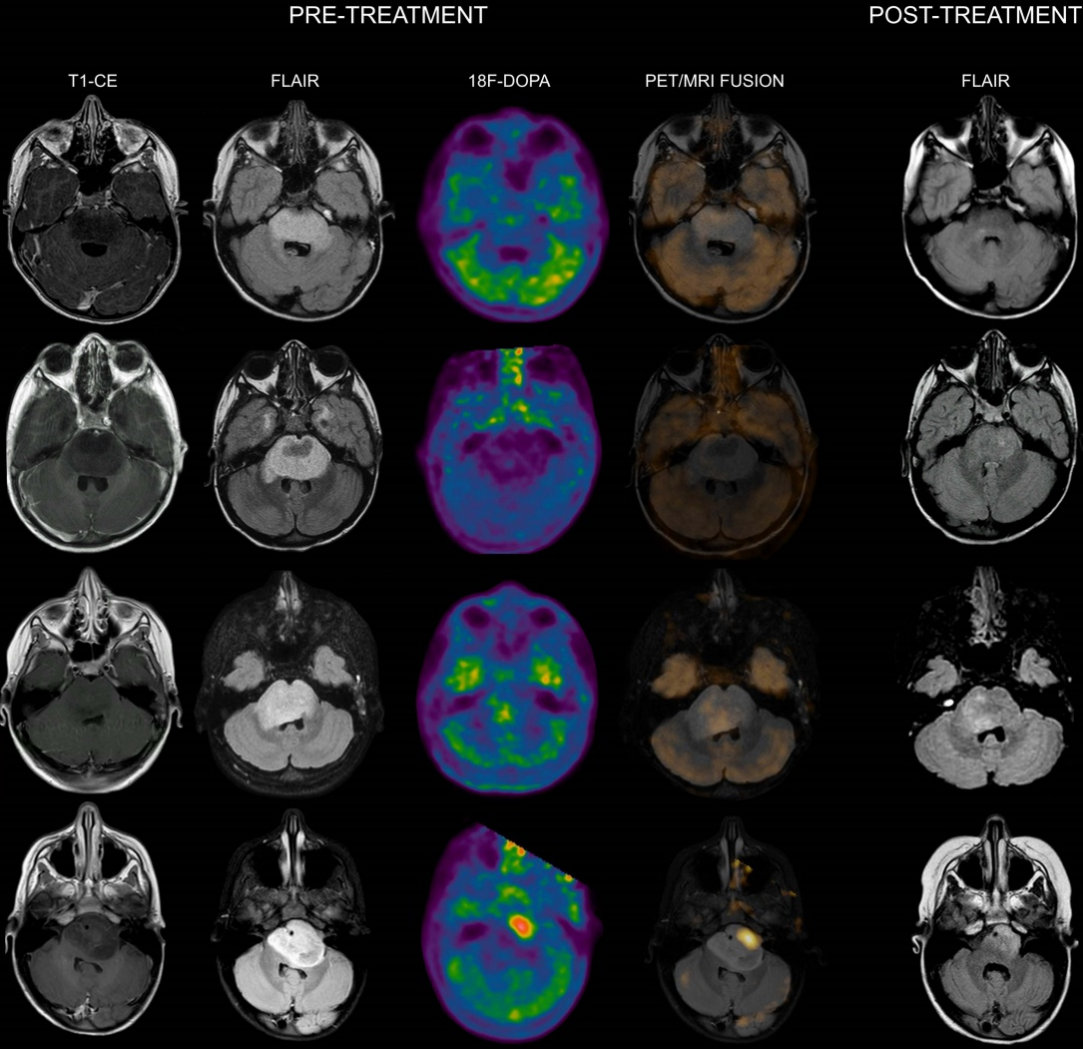
** Co-registered and fused MRI and ^18^F-DOPA PET images of DIPGs with T/S ≤ 1.** First row: 8-year-old male. Pre-treatment contrast-enhanced (CE) T1-weighted imaging did not show contrast enhancement. MRI tumor volume (as delineated on FLAIR images) was 31.46 cc. ^18^F-DOPA PET and fused ^18^F-DOPA PET/MRI revealed absence of increased tracer uptake within the lesion (T/S 0.44). Post-treatment FLAIR at maximum response demonstrated an MRI tumor volume of 7 cc. Overall survival (OS) was 27 months. Second row: 7-year-old male (diffuse astrocytoma, H3K27M wild-type). Pre-treatment CE T1-weighted imaging did not reveal areas of contrast enhancement. MRI tumor volume was 45 cc. ^18^F-DOPA PET and fused ^18^F-DOPA PET/MRI revealed absence of increased tracer uptake (T/S 0.45). On post-treatment FLAIR the MRI tumor volume was 16 cc. OS was 38 months. Third row: 7-year-old female. Pre-treatment CE T1-weighted imaging showed lack of contrast enhancement. MRI tumor volume was 25.06 cc. PET images revealed absence of increased tracer uptake in the vast majority of the lesion with small areas of mildly-moderately increased uptake (T/S 0.7). PET tumor volume was 4 cc, corresponding to a uniformity of 15.96%. Post-treatment FLAIR showed an MRI tumor volume of 8.35 cc. Notice the degree of overlap between more prominent residual components on FLAIR and increased ^18^F-DOPA uptake. OS was 16 months. Fourth row: 5-year-old female. Pre-treatment CE T1-weighted imaging did not show contrast enhancement. MRI tumor volume was 25.03 cc. ^18^F-DOPA PET and fused ^18^F-DOPA PET/MRI revealed mildly-moderately increased uptake (T/S 0.93) within the left ventrolateral component of the lesion (PET tumor volume: 8 cc, uniformity: 31.96%) corresponding to the most prominent residual component following treatment. Post-treatment FLAIR demonstrated an MRI tumor volume of 11.27 cc. OS was 28 months.

**Figure 2:**
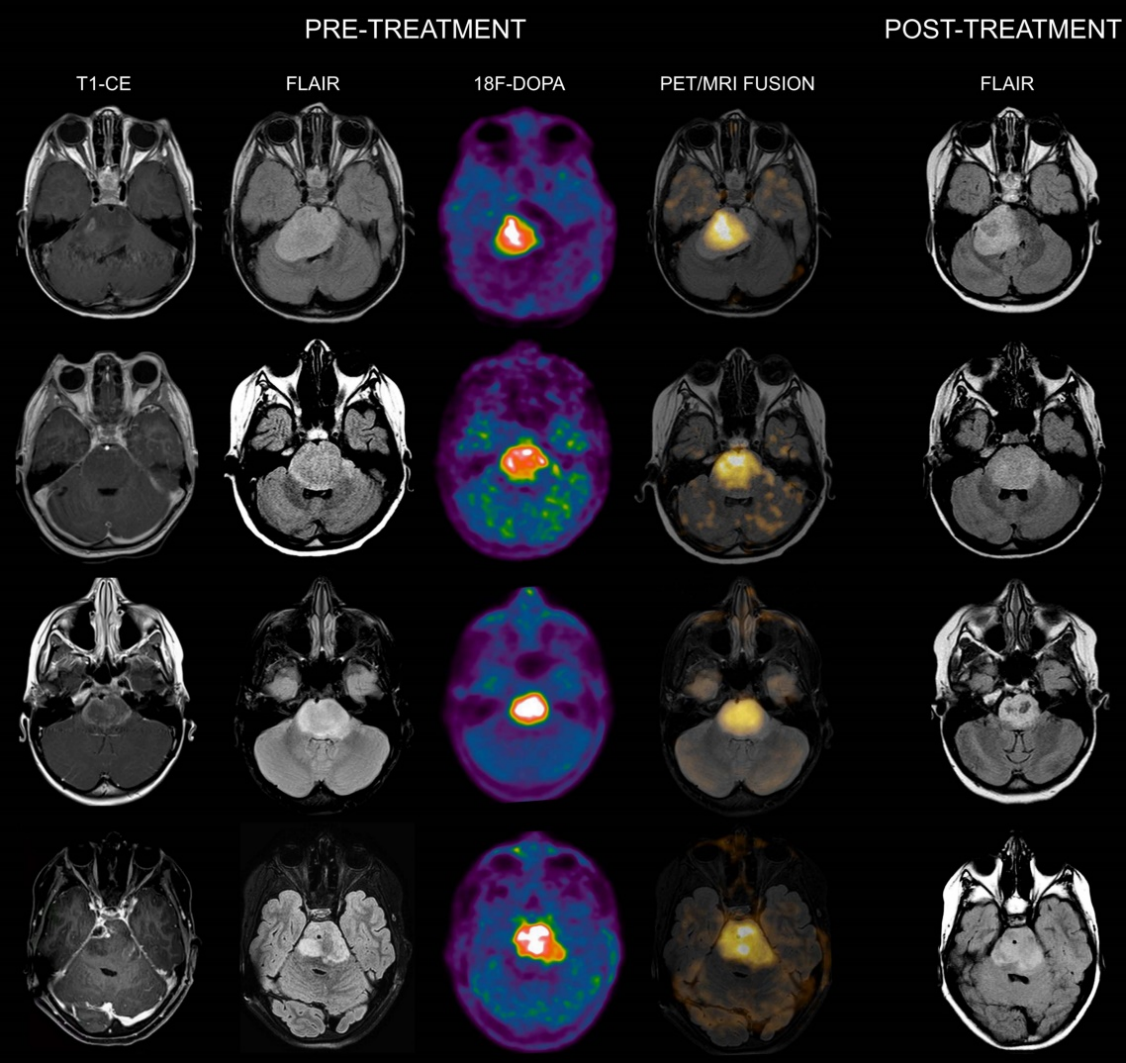
** Co-registered and fused MRI and ^18^F-DOPA PET images of DIPGs with T/S > 1.** First row: 4-year-old female. Pre-treatment contrast-enhanced (CE) T1-weighted imaging showed ring enhancement. MRI tumor volume was 40 cc. ^18^F-DOPA PET and fused ^18^F-DOPA PET/MRI revealed markedly increased uptake (T/S 1.09) with a PET tumor volume of 21 cc, corresponding to a uniformity of 52.5%. Post-treatment FLAIR demonstrated an MRI tumor volume of 28.9 cc. Overall survival (OS) was 9 months. Notice the coincidence between the tumoral component with increased uptake and the residual lesion, and the tumor volume reduction in the left ventrolateral pons corresponding to absence of increased uptake. Second row: 7-year-old male (anaplastic astrocytoma, H3K27M-mutant). Pre-treatment CE T1-weighted imaging did not reveal areas of ring enhancement. MRI tumor volume was 34.06 cc. On ^18^F-DOPA PET and fused ^18^F-DOPA PET/MRI the T/S was 1.27, the tumor volume 25 cc, and the uniformity 73.39%. Following treatment, the MRI tumor volume was of 33.7 cc. OS was 10 months. Third row: 10-year-old female. On pre-treatment CE T1-weighted imaging there was extensive ring enhancement. MRI tumor volume was 26 cc. On ^18^F-DOPA PET and fused ^18^F-DOPA PET/MRI the lesion presented a T/S of 1.7 and a tumor volume of 18 cc, corresponding to a uniformity of 69.23%. Post-treatment FLAIR image demonstrated an MRI tumor volume of 20 cc. OS was 6 months. Fourth row: 10-year-old male (glioblastoma, H3K27M-mutant). Pre-treatment CE T1-weighted imaging revealed a right focal area of ring enhancement. MRI tumor volume was 46 cc. ^18^F-DOPA PET and fused ^18^F-DOPA PET/MRI demonstrated markedly increased uptake (T/S 2.32) and a tumor volume of 31 cc, corresponding to a uniformity of 67.39%. Following treatment, the MRI tumor volume was 42 cc. OS was 6 months.

When evaluating the association between ^18^F-DOPA uptake tumor volume at admission with the MRI tumor volume at maximum response, areas of increased tracer uptake corresponded to regions with more prominent residual components/lack of response following treatment in all patients. Representative images of DIPGs at admission and following first-line ChT-RT are reported in **Figures 1 and 2.**

### Prognostic evaluation

The median OS of the whole cohort was 10 months (range 5-38 months) and cumulative probability of survival (OS) at 12 months was 32% (95% confidence interval [CI]: 13% - 52%).

**Figure 3:**
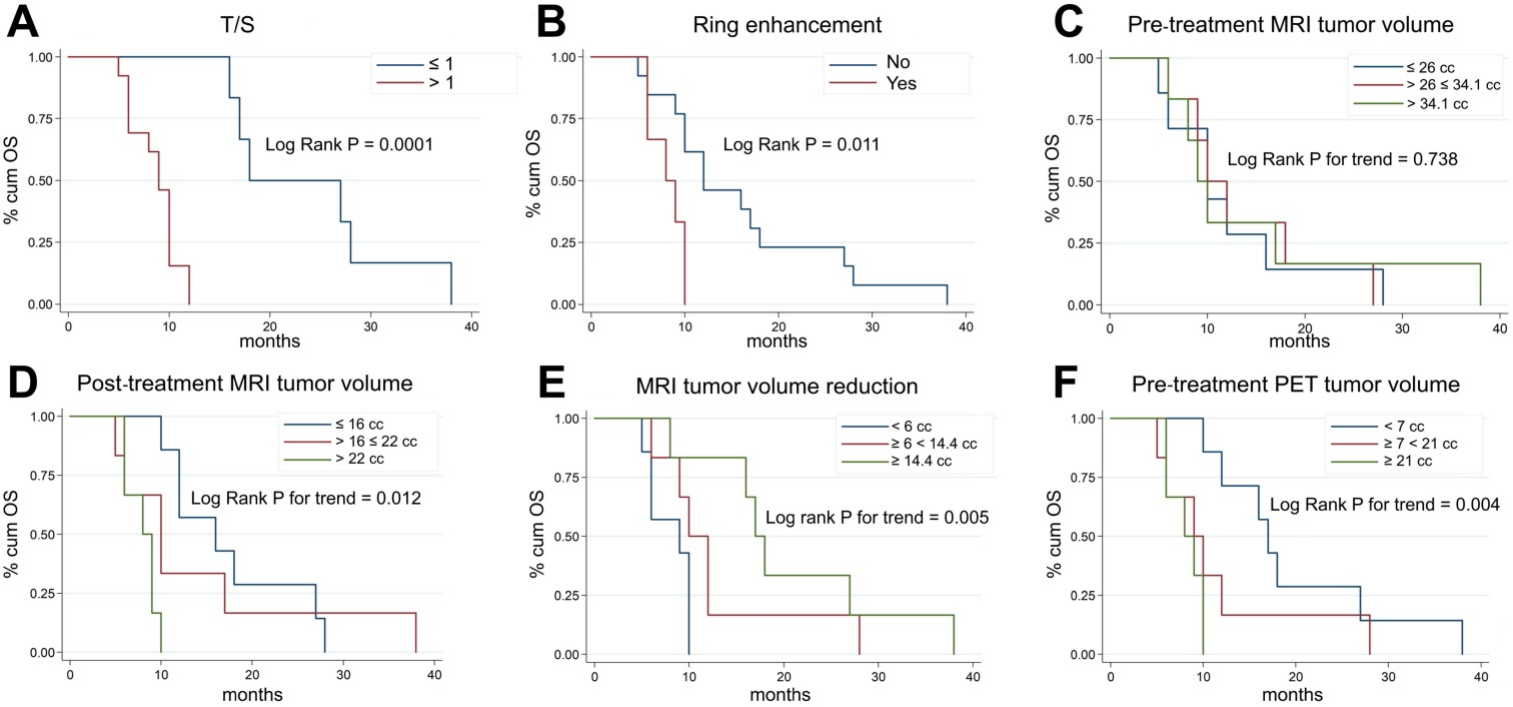
Kaplan-Meier plots of overall survival (OS) according to T/S (A), ring enhancement (B), pre-treatment MRI tumor volume (C), post-treatment MRI tumor volume at maximum response (D), degree of MRI tumor volume reduction (E), and pre-treatment ^18^F-DOPA PET tumor volume (F). ^18^F-DOPA PET and MRI (based on FLAIR images) tumor volume measurements were categorized considering tertiles of each distribution.

**Figure 3** shows Kaplan-Meier OS curves for all the main risk factors analyzed in our study. Subjects with T/S ratios > 1 (markedly increased uptake) had a significantly higher risk of death (*p =* 0.0001, **Figure 3A**) than those with T/S ≤ 1. At the same time, we found that at univariate level, H3K27M-mutant lesions and patients with ring enhancement on post-contrast T1-weighted images had a significantly lower survival (hazard ratio [HR] = 3.51, 95% CI: 1.11-11.1, *p =* 0.024, not shown, and HR = 3.91, 95% CI: 1.17-13.1, *p =* 0.011, **Figure 3B**). Patients with larger MRI tumor volumes at admission did not show lower survival (*p =* 0.738, **Figure 3C**). By contrast, post-treatment MRI tumor volume at maximum response and tumor volume reduction following treatment were significantly correlated with OS (*p =* 0.012, **Figure 3D**, and *p =* 0.005, **Figure 3E**). Median age and gender were not correlated with OS (*p =* 0.608, not shown), whereas patients with greater ^18^F-DOPA PET tumor volume (> 21 cc *vs.* < 7 cc: HR = 5.74, 95% CI: 1.46-22.62; 7-21 cc *vs.* < 7 cc: HR = 2.31, 95% CI: 0.73-7.34, *p =* 0.004, **Figure 3F**) and uniformity (*p =* 0.001, not shown) were at higher risk of death. However, when we adjusted risk estimates for age and gender by using a multivariate Cox model, including only the covariates with a significant association on univariate analysis, only T/S (HR = 55.50, 95% CI: 5.3-580.3, *p =* 0.001), ring enhancement (HR 12.4, 95% CI: 1.8-83.1, *p =* 0.01) and MRI tumor volume reduction following treatment (HR 0.1, 95% CI: 0.02-0.56, *p =* 0.01) resulted to be significant predictors of OS. A trend emerged for age (*p =* 0.061) (**Table 2**).

## Discussion

The aim of this study was to evaluate and correlate ^18^F-DOPA uptake intensity and extent (PET tumor volume and uniformity) with conventional MRI indices, and with treatment response and survival of pediatric patients with DIPGs. To the best of our knowledge, no prior studies have so far evaluated the role of ^18^F-DOPA PET on a selected population of children with DIPGs. Overall, a limited number of studies have assessed PET imaging in DIPGs 21,22,24-27.

Zukotynski et al. 21 analyzed the role of ^18^F-fluorodeoxyglucose (FDG) PET in patients with newly diagnosed DIPGs and observed that the intensity of FDG uptake did not correlate with outcome; however, in patients with ^18^F-FDG PET uptake involving more than half of the tumor, survival appeared to be decreased. A more recent study from the same group 24 evaluated ^18^F-FDG histogram metrics in children with DIPGs, revealing that parameters such as skewness or kurtosis did not show a significant association with outcome.

An additional multimodal study with ^18^F-FDG PET performed by Goda et al. 25 did not find a significant correlation between baseline DIPGs ^18^F-FDG uptake and survival, even though patients with increased PET uptake had a lower 1-year OS (40%) and PFS (33%) compared to patients with lower uptake (66.7% and 40%, respectively).

Of note, among amino-acid PET tracers, only three prior pediatric research studies, all performed with ^11^C-Methionine (MET), have been performed 22,26,27. Two of these studies evaluated both ^18^F- FDG and ^11^C-MET to characterize the tumoral metabolic activity. In particular, Pirotte et al. 26 investigated a group of children with different infiltrating brainstem tumors highlighting the role of PET imaging to biopsy planning and target selection. Rosenfeld et al. 27, a few years later, found that patients with the shortest survival time were those who had ^18^F-FDG negative and ^11^C-MET positive scans, whereas patients with the longest survival times were those with both negative ^18^F-FDG and ^11^C-MET scans suggesting a trend toward improved survival, even though the correlation was not statistically significant.

A very recent study performed by Tinkle et al*.* 22 evaluated the role of ^11^C-MET PET in 22 subjects with typical and atypical DIPGs, treated with RT using conventional fractionation (1.8Gy per day) to a total dose of 54‐55.8Gy; although baseline ^11^C-MET PET intensity and uniformity metrics did not correlate with survival outcomes, initial ^11^C-MET avidity overlapped with recurrent tumor in 100% of cases, delineating regions at increased risk for recurrence. These authors also suggested to explore the role of ^18^F-DOPA in this pediatric tumor subtype.

In the present study, a significant relationship between ^18^F-DOPA uptake avidity and extent (^18^F-DOPA tumor volume and uniformity) was found in terms of OS on univariate analysis, and the ^18^F-DOPA PET metric T/S was an independent predictor of survival on multivariate analysis. Of note, all DIPGs with a T/S ratio >1 (markedly increase tracer uptake) presented an OS ≤ 12 months. As demonstrated in prior studies 16-18,23, T/S ratio is an extremely helpful ^18^F-DOPA PET parameter to predict pediatric diffuse astrocytic tumor behavior in a clinical diagnostic setting; moreover, ^18^F-DOPA uptake in the striatum allows to further stratify tumor uptake ratios when compared to other amino-acid PET tracers. Of note, even though striatal ^18^F-DOPA uptake may be subject to changes related to age in adults 28,29, we believe that in our pediatric population (children aged 3 to 10 years) the impact of age-related striatal uptake does not appear to be significant.

As previously reported in a large DIPG study 4, also in our current research the presence of ring enhancement on MRI proved to be an additional independent predictor of adverse OS. Of note, when considering our population, only 6 DIPGs presented this MRI finding while, among the remaining 13 subjects without ring enhancement, 7 additional patients presented an OS ≤ 12 months. Overall, among subjects with an OS ≤ 12 months, 46% presented ring enhancement whereas 100% presented a T/S ratio >1. Regarding the relationship between ^18^F-DOPA accumulation and areas of contrast-enhancement, in all but one subjects markedly increase uptake extended beyond these areas, and was present in additional seven subjects without any area of contrast-enhancement, thus providing additional non-invasive diagnostic information in keeping with prior studies 15,16,30.

Tumor volume reduction following therapy (difference between MRI tumor volume at admission and at maximum response following first line treatment) also correlated significantly with OS on multivariate analysis. This result is in line with a prior study demonstrating that patients with more substantial tumor volume decrease following therapy had better OS 20. Differently from the above-mentioned study, MRI tumor volume at admission did not show a significant correlation with outcome.

When compared to MRI tumor volume decrease, the main potential advantage of ^18^F-DOPA PET T/S ratio or presence of ring enhancement consists in the possibility of obtaining prognostic information at admission and not following treatment.

When compared to the amino-acid PET tracer ^11^C-MET, which did not show a correlation between uptake and outcome 22, our findings suggest that in DIPGs ^18^F-DOPA uptake might be related to additional mechanisms, such as expression of supplementary amino-acid transporters, as demonstrated in a prior study where ^18^F-DOPA uptake values of brain gliomas were not exclusively dependent on L-type amino-acid transporter (LAT) 1 expression 31,32. In adult diffuse gliomas has been suggested that an apparent discrepancy between the uptake patterns of ^11^C-MET and ^18^F-DOPA may be linked to the metabolomic profile of IDH-mutated tumors 33. Since pediatric diffusely infiltrating astrocytic tumors typically do not present IDH mutation different mechanisms should be considered. In a current ongoing unpublished research, our preliminary findings show that the glutamine transporter sodium-coupled neutral amino-acid transporter (SNAT) 1 (Slc38a1) is involved in ^18^F-DOPA uptake of cell lines of pediatric high-grade gliomas, in addition to LAT1. Further studies are needed to evaluate and elucidate the potential role of glutamine for DIPGs survival and proliferation. Glutamine addiction in adult high-grade gliomas is well known 34.

In a recent study we also demonstrated that anaplastic astrocytomas and glioblastomas H3K27M-mutant arising in midline brain structures had a significant higher uptake than wild-type midline high-grade gliomas 18. H3K27M-mutant diffuse midline gliomas, which include about 85% of DIPGs, have been reported to exhibit elevated expression and dependency on dopamine receptor (DR) D2 35. DRD2 is a G protein-coupled receptor that promotes tumor growth. A recent study also demonstrated that glioblastoma cells (including tumor samples from patients) can synthesize and secrete dopamine, suggesting an autocrine signaling process and an underlying role for dopamine in gliomagenesis 36. DOPA is a precursor for dopamine synthesis, and when compared to other amino-acid PET tracers ^18^F-DOPA uptake might be more specifically related to cells that synthesize and secrete dopamine.

In the study performed by Tinkle et al. 22 ^11^C-MET PET-delineated tumor extended beyond the T2-FLAIR abnormality in most patients at diagnosis. This is in distinction to our findings where ^18^F-DOPA PET-delineated tumor was always confined within the MRI tumor volume. However, when comparing the association between pre-treatment ^18^F-DOPA PET tumor volume and post-treatment MRI tumor volume, areas of increased ^18^F-DOPA uptake corresponded to regions with more prominent residual components/lack of response in all patients. This finding is in line with the above-mentioned study 22 demonstrating a correlation between the location of increased ^11^C-MET uptake at admission and the subsequent region of local tumor progression.

Considering the significant association between ^18^F-DOPA uptake avidity and extent with outcome, our findings indicate that tumoral areas with increased tracer uptake show a more aggressive biological behavior, with reduced sensitivity to ChT-RT.

In the era of precision medicine, the results of the present study could delineate subcategories of patients who might benefit from personalized management strategies.

Given the lack of current medical therapies able to significantly impact outcome and the known feasibility of re-irradiation for recurrent DIPGs 37,38, a more personalized RT treatment might be tested on the basis of the metabolic characteristics of the lesion; such an approach might also be considered as selection criteria in discriminating DIPGs that may better benefit re-irradiation, since not all DIPGs improve prognosis with re-RT, as reported in a recent meta-analysis 37.

In addition, patients with lack of increased ^18^F‐DOPA uptake or with mild-moderate uptake (which correlate with higher tumor volume response degree and better outcome) might be selected to perform a first radiotherapy course with lower dose than 54 Gy at admission, in order to give them the possibility of multiple radiotherapy courses. A current clinical trial (NCT03620032; clinicaltrials.gov) is actively investigating multiple elective RT courses in DIPGs.

The role of ^18^F-DOPA PET might also be evaluated on follow-up studies to assess treatment response. A prior study performed in adult patients with recurrent high-grade malignant gliomas on antiangiogenic treatment demonstrated the powerful role of ^18^F-DOPA PET in assessing treatment response and for providing prognostic information 39.

Our results should be interpreted with awareness of some limitations. This is a retrospective study in which a limited number of patients was evaluated; however, we included only pediatric patients with DIPGs studied with ^18^F-DOPA PET, and currently our cohort constitutes to the best of our knowledge the first and largest series of DIPGs evaluated with this technique so far. We acknowledge that only 10 out of 19 subjects underwent biopsy, thus limiting the molecular characterization of the entire cohort. However, the aim of the present study was to evaluate the degree of correlation between ^18^F-DOPA PET avidity and extent with outcome, and molecular characterization of DIPG did not influence the treatment regimen which was homogeneous among subjects. In the current study we evaluated only static ^18^F-DOPA PET parameters; as highlighted in a recent research on adult gliomas 40, dynamic ^18^F-DOPA PET analysis in pediatric gliomas might provide additional information in future investigations. Furthermore, in this study we did not compare ^18^F-DOPA PET with advanced MR imaging modalities; as highlighted in a recent study which demonstrated a significant association of different advanced MR imaging metrics with survival 41, prospective clinical trials should be performed in order to evaluate the synergic role of these techniques. We recognize that in the current study we did not evaluate the prognostic role of T/N, even though we used this parameter to stratify tumor uptake patterns and to delineate tumor volume. However, in pediatric diffusely infiltrating astrocytic tumors, according to our experience, the T/N ratio did not provide additional information when compared to T/S, in terms of differentiation between low-grade and high-grade lesions and with regard to outcome prediction 17,23; of note, in pediatric diffuse midline gliomas, which according to the revised 2016 World Health Organization classification of tumors of the central nervous system include DIPGs, T/S was the only parameter able to discriminate H3K27M-mutant from wild-type tumors, independently of histology 18. Furthermore, as reported by the Joint EANM/EANO/RANO practice guidelines/SNMMI procedure standards for imaging of gliomas using PET with radiolabelled amino-acids 42, the striatum is the only recommended reference region for semiquantitative measures of ^18^F-DOPA PET activity. However, regarding cut-off thresholds for definition of biological tumor volume the EANM/RANO/EANO and SNMMI guidelines indicate for ^18^F-DOPA a standardized uptake value (SUV) higher than the mean SUV in healthy striatum 39,42. Since it is acknowledged that this cut-off value lacks histological validation 42, and according to Cicone et al. 43, we used a different threshold. Of note, since when compared to their adult counterparts, pediatric astrocytic tumors are considered biologically distinct entities 44, and in light of the complex physiological changes occurring in the brain during development, especially in the first two years of life (i.e., cortical organization, white matter myelination, changes in cerebral blood flow), current adults guidelines and ^18^F-DOPA PET literature based on adult gliomas may not be entirely applicable to pediatric gliomas, especially in the era of precision medicine.

## Conclusions

^18^F-DOPA PET imaging provides useful non-invasive information for evaluating the metabolism of DIPGs. Our results demonstrate that the ^18^F-DOPA PET metric T/S is an independent predictor of overall survival. ^18^F-DOPA uptake extent corresponds to tumoral components with a more aggressive biological behaviour, less sensitive to first line ChT-RT. Larger prospective studies are needed to evaluate whether ^18^F-DOPA PET can provide additional information for more personalized management schemes.

## Supplementary Material

Supplementary table S1.

## Figures and Tables

**Table 1:** Summary of patient characteristics, imaging findings and survival

Patient characteristics and imaging findings	n (%)	Median (range)
**Gender**		
Male	7 (37%)	
Female	12 (63%)	
**Age at diagnosis (years)**		
<5	3 (16%)	7 (3-10)
5-8	12 (63%)	
>8	4 (21%)	
**Histological and molecular data**		
DA, H3K27M-wt	1 (5%)	
AA, H3K27M-wt	1 (5%)	
AA, H3K27M-m	4 (21%)	
GB, H3K27M-m	4 (21%)	
ND	9 (48%)	
**Ring enhancement**		
Y	6 (31.6%)	
N	13 (68.4%)	
**^18^-F-DOPA uptake patterns**		
T/N≤1	4 (21%)	T/S 0.44 (0.24-0.55)
T/N>1-T/S≤1	2 (10.5%)	T/S 0.81 (0.70-0.93)
T/S >1	13 (68.5%)	T/S 1.27 (1.06-2.32)
**Imaging findings and OS according to PET semi-quantification**		
***Pre-treatment MRI tumor volume (cc)***		27.64 (18.58-55.63)
T/N≤1	39.51 (31.46-55.63)	
T/N>1-T/S≤1	25.04 (25.03-25.06)	
T/S >1	27.21 (18.58-46.35)	
***Post-treatment MRI tumor volume (cc)***		20 (7-42)
T/N≤1	13.77 (7-20.54)	
T/N>1-T/S≤1	9.81 (8.35-11.27)	
T/S >1	22 (11.18-42)	
***Pre-treatment PET tumor volume (cc)***		17.2 (0.86-31)
T/N≤1	No increased uptake	
T/N>1-T/S≤1	6 (4-8)	
T/S >1	18 (0.86-31)	
***Pre-treatment PET uniformity (%)***		52.5 (3.94-92.57)
T/N≤1	No increased uptake	
T/N>1-T/S≤1	23.96% (15.96-31.96%)	
T/S >1	62.56% (3.94-92.57%)	
**Overall survival (months)**		10 (5-38)
T/S≤1	23 (16-38)	
T/S >1	9 (5-12)	

*DA:* diffuse astrocytoma, *AA:* anaplastic astrocytoma, *GB:* glioblastoma, *wt:* wildtype, *m:* mutant, *ND:* not done, *Y:* yes, *N:* no.

**Table 2:** Cox regression multivariate analyses (subjects n=19)

Endpoint	Parameter	HR*	95% CI	*p*
OS	T/S			
	≤ 1	1.0	5.3-580.3	0.001
	> 1	55.5		
	**Ring enhancement**			
	N	1.0	1.8-83.1	0.01
	Y	12.4		
	**Tumor volume reduction**			
	≤ 11.1 (median)	1.0	0.02-0.56	0.01
	> 11.1	0.1		
	**Age**	0.7	0.5-1.0	0.061

*Hazard ratios (HR) are adjusted for age and sex; 95% CI: 95% confidence interval, OS: overall survival. 1/VIF (tolerance): T/S = 0.794; Ring enhancement = 0.808; Tum. vol. red. = 0.762; age = 0.834.

## Abbreviations

PET: positron emission tomography
MRI: magnetic resonance imaging
DOPA: dihydroxyphenylalanine
DIPG: diffuse intrinsic pontine glioma
OS: overall survival
T/S: tumor/striatum
RT: radiotherapy
ChT: chemotherapy
FLAIR: fluid attenuation inversion recovery
CT: computed tomography
FDG: fluorodeoxyglucose
MET: methionine
LAT: L-type amino acid transporter
SNAT: sodium-coupled neutral amino acid transporter
DR: dopamine receptor
