# Supplementary material for: Correlation of multimodal 18F-DOPA PET and conventional MRI with treatment response and survival in children with diffuse intrinsic pontine gliomas
Source: Theranostics. 2020 Oct 25;10(26):11881–91. doi: 10.7150/thno.50598 (PMC7667677; doi:10.7150/thno.50598)
Supplement: Supplementary file 1 — Supplementary table S1. [file thnov10p11881s1.pdf]

Table S1. Detailed report of patient characteristics, imaging findings and survival

Case	Age at diagnosis	Sex	Histological and molecular diagnosis	Ring enhancement (Y/N)	T/S	T/N	Pre-treatment MRI tumor volume (cc)	Pre-treatment PET tumor volume (cc)	Post-treatment MRI tumor volume (cc)	OS
1	3	M	AA, H3K27M-m	N	1.50	2.20	18.58	17.20	17.90	5
2	3	F	ND	N	0.24	0.47	34.03	no increased uptake	11.49	18
3	4	F	ND	Y	1.09	2.09	40.00	21.00	28.90	9
4	5	M	ND	N	1.24	1.76	27.21	7.50	16.00	10
5	5	F	ND	Y	1.10	1.71	22.78	5.00	21.25	10
6	5	F	ND	N	0.93	1.62	25.03	8.00	11.27	28
7	6	F	GB, H3K27M-m	Y	1.55	2.85	46.35	29.00	32.00	8
8	6	F	ND	N	1.06	1.89	21.80	0,86	11.18	12
9	6	F	ND	N	0.55	0.95	55.63	no increased uptake	20.54	17
10	7	F	AA, H3K27M-m	N	1.48	2.73	27.64	22.00	22.12	6
11	7	M	AA, H3K27M-m	N	1.27	2.18	34.06	25.00	33.70	10
12	7	M	GB, H3K27M-m	Y	1.10	1.91	24.03	21.00	22.00	10
13	7	F	ND	N	0.70	1.10	25.06	4.00	8.35	16
14	7	M	DA, H3K27M-wt	N	0.45	0.75	45.00	no increased uptake	16.00	38
15	8	M	AA, H3K27M-wt	N	0.44	0.67	31.46	no increased uptake	7.00	27
16	9	F	AA, H3K27M-m	N	1.22	2.49	26.69	13,91	15.08	12
17	10	M	GB, H3K27M-m	Y	2.32	3.78	46.00	31.00	42.00	6
18	10	F	GB, H3K27M-m	N	1.73	2.52	27.77	8,61	26.00	9
19	10	F	ND	Y	1.70	2.89	26.00	18.00	20.00	6

OS overall survival, *M* male, *F* female, *DA* diffuse astrocytoma, *AA* anaplastic astrocytoma, *GB* glioblastoma, *wt* wildtype, *m* mutant, *ND* not done, *Y* yes, *N* no
